# Adenylosuccinate acts as a novel pro-angiogenic factor

**DOI:** 10.21203/rs.3.rs-10221033/v1

**Published:** 2026-07-27

**Authors:** Brennon Schofield, Drew R. Seeger, Derek Besch, Peddanna Kotha, Svetlana A. Golovko, Bryon D. Grove, Anahita Mansouripour, Meredith Parmer, Shahram Solaymani-Mohammadi, Mikhail Y. Golovko

**Affiliations:** Department of Biomedical Sciences, School of Medicine and Health Sciences, University of North Dakota, Grand Forks, ND, USA

**Keywords:** Ischemia, Stroke, Brain, Angiogenesis, Adenylosuccinate

## Abstract

We recently demonstrated a dramatic 77-fold increase in brain adenylosuccinate (AdSucc) concentration during ischemia, reaching ~0.56 mM levels. Previously, the role of AdSucc induction under decreased energy charge was attributed to the activation of purine nucleotide (PNC) and the tricarboxylic acid cycle (TCA). However, careful analysis of metabolomic alterations in the ischemic brain under decreased energy charge did not confirm this role. To begin addressing the additional biological significance of the increased AdSucc under low-energy conditions associated with ischemia, we tested its angiogenic properties. Using a mouse *in vivo* angiogenesis assay, we discovered that AdSucc, but not its metabolites such as adenosine or AMP accumulated during the assay, promotes angiogenesis *in vivo* at concentrations found in ischemic tissues. Further, using *in vitro* angiogenesis assay with human umbilical vein endothelial cells (HUVEC), we demonstrated that AdSucc promotes the formation of capillary-like structures (tubes) on extracellular matrix support. We also report, for the first time, that AdSucc increases cAMP levels, supporting its signaling role, which may be linked to pro-angiogenic mechanisms. Together, our data indicate a novel mechanism of angiogenesis regulation, in which AdSucc may serve as a new signaling molecule under low-energy conditions. Further studies are required to confirm the role of endogenously produced AdSucc in tissue angiogenesis as an adaptation mechanism to low-energy conditions and repair after injury.

## Introduction

Adult angiogenesis ensures that the energetic demands of tissue are met during metabolic changes and depending on the availability of circulating oxygen and nutrients. Angiogenesis is also critically important for tissue recovery after injury. It is well documented that recovery from cerebral and myocardial infarction and traumatic injury involves angiogenesis as part of the repair process ^1,2 3–11^. Given the importance of adult angiogenesis in cerebral and cardiovascular disease and physiological adaptation to changes in O_2_ and nutrient supply, as well as its pathological role in cancer and age-related degeneration, a deeper mechanistic understanding of adult angiogenesis is warranted.

Recently, we discovered a dramatic 77-fold increase in brain adenylosuccinate (AdSucc) concentration under ischemia, which reached 0.555±0.036 nmol/mg of tissue wet weight (ww, ~0.56mM) ^12^. Previously, the significance of AdSucc increase under energy depletion caused by brain stimulation or muscle exercise was explained through activation of the purine nucleotide cycle (PNC) to support the tricarboxylic acid cycle (TCA) ^13,14^. According to this mechanism, aspartate is deaminated to fumarate (Fum) in PNC, and Fum enters TCA, thus increasing its oxidative capacity and tissue energy production ^15,16^. However, careful examination of metabolome alterations in the ischemic brain did not confirm the role of AdSucc induction in PNC and TCA activation ^12^.

In the present study, to begin addressing AdSucc alternative roles in ischemic tissues characterized by decreased energy charge, we tested the hypothesis that increased AdSucc may serve as a metabolic signal of decreased energy status and activate an angiogenic response as a compensatory mechanism. We tested AdSucc angiogenic effect in two distinct models, including *in vivo* and *in vitro* assays, and validated its signaling properties by measuring the effect of AdSucc on a secondary messenger cAMP.

## Results and Discussion

**Figure 1.**
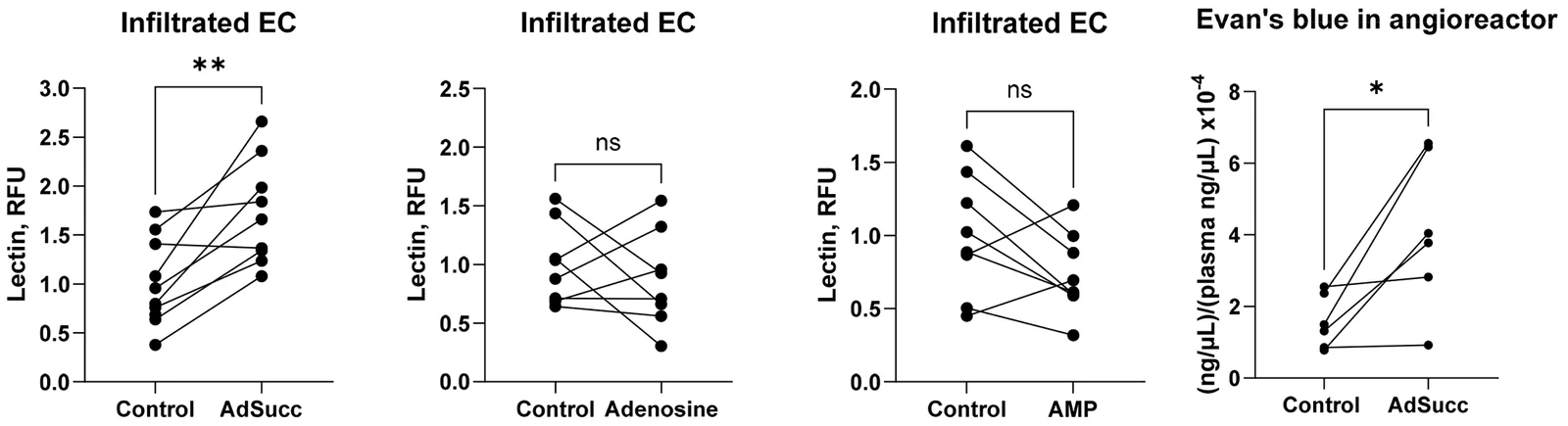
Adenylosucinnate (AdSucc) promotes angiogenesis in the Directed *In Vivo* Angiogenesis Assay (DIVAA) Silicone angioreactors containing 20 μL of VitroGel hydrogel matrix with PBS (control), AdSucc (500 μM), or accumulated products of AdSucc degradation adenosine (0.6 μM) or AMP (20 μM) were subcutaneously implanted in the contralateral flanks of RAG1^−/−^ mice. After 10 days, infiltrating endothelial cells were labeled with FITC-labeled lectin and quantified by fluorescence. A separate set of mice was perfused with Evan’s blue to measure functional perfusion. Data are expressed as relative fluorescent units (RFU) for endothelial cell infiltration, and as normalized to plasma Evan’s blue concentration for angioreactor perfusion. * - significantly different (*p*<0.05), ** - significantly different (*p*<0.01) as calculated using two-tailed, paired Student’s *t* test. Number of biological replicates *n*=9 for AdSucc treatment, *n*=8 for adenosine and AMP, and *n*=6 for perfusion experiments.

**Figure 2.**
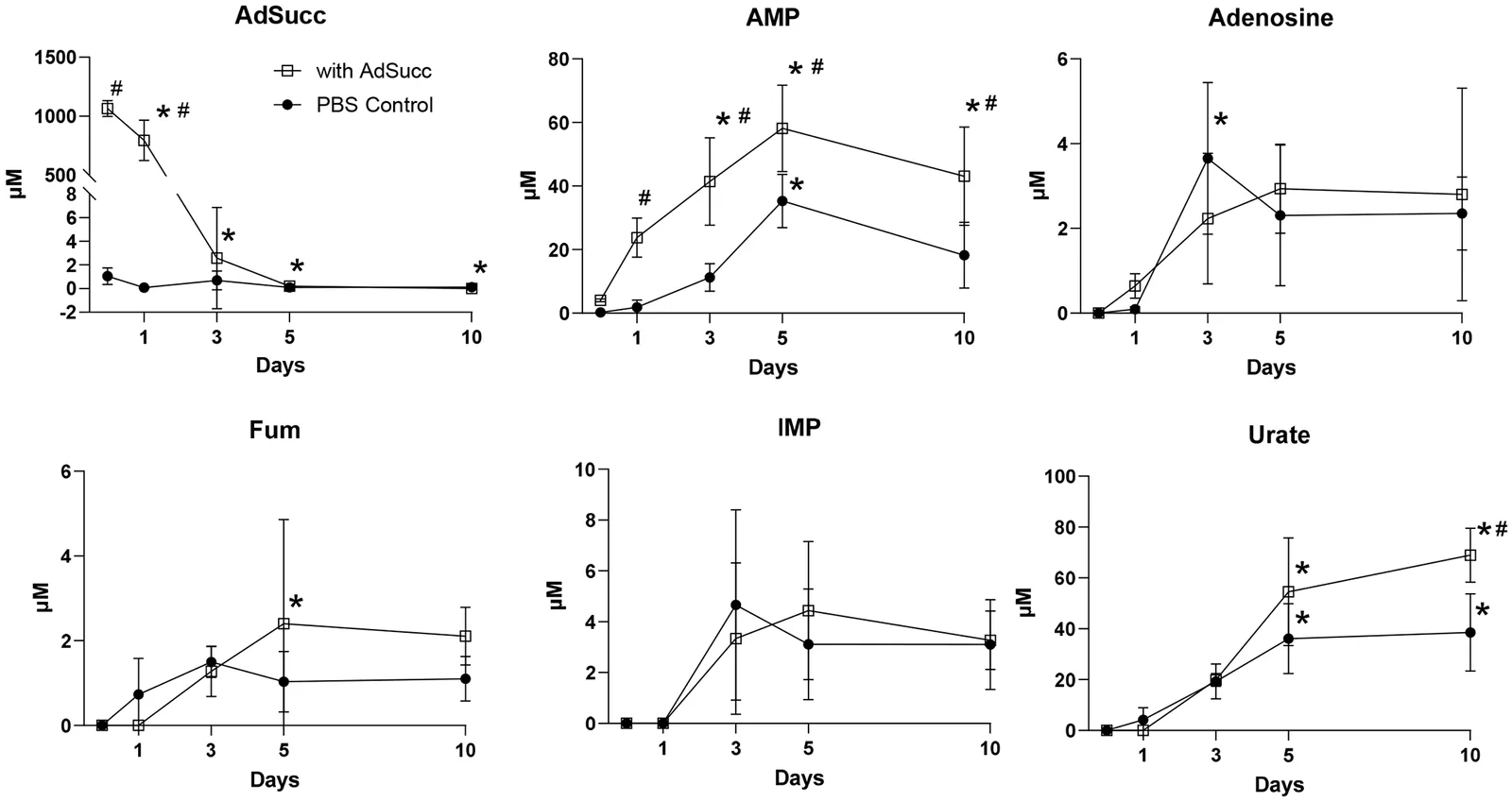
Analysis of adenylosuccinate (AdSucc) metabolites in implanted DIVAA angioreactors. Silicone angioreactors containing 20 μL of VitroGel hydrogel matrix with PBS (control) or AdSucc (500 μM) were implanted in Rag−/− mice for 0 (not implanted), 1, 3, 5, or 10 days, as described. Upon removal, metabolites were analyzed using UPLC-MS^e^ against a stable isotope-labeled internal standards. Data are presented as individual values and mean±standard deviation (*n*=4). Statistical differences found between AdSucc and PBS containing angioreactors at the same time point are denoted with (#), and differences from the 0-time point are denoted with (*) using a one-way ANOVA with Tukey’s *post hoc* test (*p*<0.05).

Our *in vivo* DIVAA data demonstrate that the number of infiltrated endothelial cells was significantly increased by 1.8-fold in angioreactors containing AdSucc at day 10 after implantation compared to control angioreactors (Fig. 1), indicating a strong pro-angiogenic effect of AdSucc. Importantly, the initial AdSucc concentration (1mM) was close to the levels found in ischemic tissues ^12^. We did not continue the assay beyond 10 days because AdSucc concentrations decreased to levels similar to those in the control-implanted angioreactors at day 3 (Fig. 2).

Because an increased number of infiltrated endothelial cells does not necessarily indicate an increase in perfused vessels, we measured the effect of AdSucc on blood volume in angioreactors. Using Evan’s blue dye, which binds to albumin and does not penetrate through capillaries under normal conditions ^17,18^, we found a significant 2.9-fold increase in the normalized Evan’s blue dye concentration in angioreactors containing AdSucc (Fig. 2). Although these data might indicate an increased leakage of newly formed vessels in the presence of AdSucc, together with increased endothelium cell number, they support pro-angiogenic effect of AdSucc in this *in vivo* assay.

It is possible that products of AdSucc degradation, rather than AdSucc *per se*, exert the observed pro-angiogenic effect. It is well accepted that AdSucc is rapidly degraded to Fum and adenosine (Ade) monophosphate (AMP) by adenylosuccinate lyase ^19,20^. AMP might directly exert pro-angiogenic effect through an AMP- activated protein kinase signaling cascade ^21^ or by cross-activating adenosine receptors ^22,23^. Fum might also stimulate angiogenesis by stabilizing hypoxia-inducible factors ^24^, a strong pro-angiogenic regulators ^25^. AMP is further dephosphorylated to Ade by 5’-nucleotidases such as CD73 or cytosolic isoforms ^26^, which activates P1 purinergic receptors with a resultant strong stimulation of angiogenesis ^27^. Alternatively, AMP might be deaminated by AMP deaminases, leading to the production of inosine monophosphate (IMP) ^28^, or oxidized to form uric acid as a final catabolic product ^29^. These AdSucc metabolites may affect cellular growth, migration, and adhesion, thereby altering angiogenesis upon AdSucc treatment.

Considering the pro-angiogenic role of AdSucc degradation products, we next performed metabolomics analysis of the hydrogel matrix containing vehicle or AdSucc at different time points after implantation (Fig. 2) and validated their contribution to the observed AdSucc effect on angiogenesis (Fig. 1). AdSucc was progressively decreased after angioreactor implantation, and after day 3 was not significantly different from control angioreactors. The AdSucc decrease might result from washout via diffusion, or from enzymatic degradation. AMP, the major product of AdSucc degradation through lyase reaction, was higher at all time points after transplantation, with an average difference of 20 μM between control and AdSucc-loaded angioreactors. Initial AMP accumulation might be attributable to AdSucc degradation. However, the increased levels in AdSucc-containing angioreactors after day 3 might be due to enhanced cell infiltration driven by potentiated angiogenesis, as AdSucc did not differ between conditions after day 3, and AMP is mainly localized intracellularly. Adding this amount of AMP (20 μM) did not produce an angiogenic effect (Fig. 1). Though Ade was not significantly different between conditions at the same time points (Fig. 2), considering its potent pro-angiogenic effect, we tested the contribution of 0.6 μM of Ade to the AdSucc angiogenic effect, which is the highest difference in Ade concentration between conditions at the same time points. Similar to AMP, it did not have an angiogenic effect at the tested levels (Fig. 1). IMP and Fum were not different between AdSucc-loaded and control angioreactors at any of the time points, while urate was higher on day 10 only, probably becuase of increased cell number associated with potentiated angiogenesis in the AdSucc angioreactors (Fig1 and 2). Together, these data indicate that AdSucc pro-angiogenic effect is independent of its products of degradation under the assay conditions.

**Figure 3.**
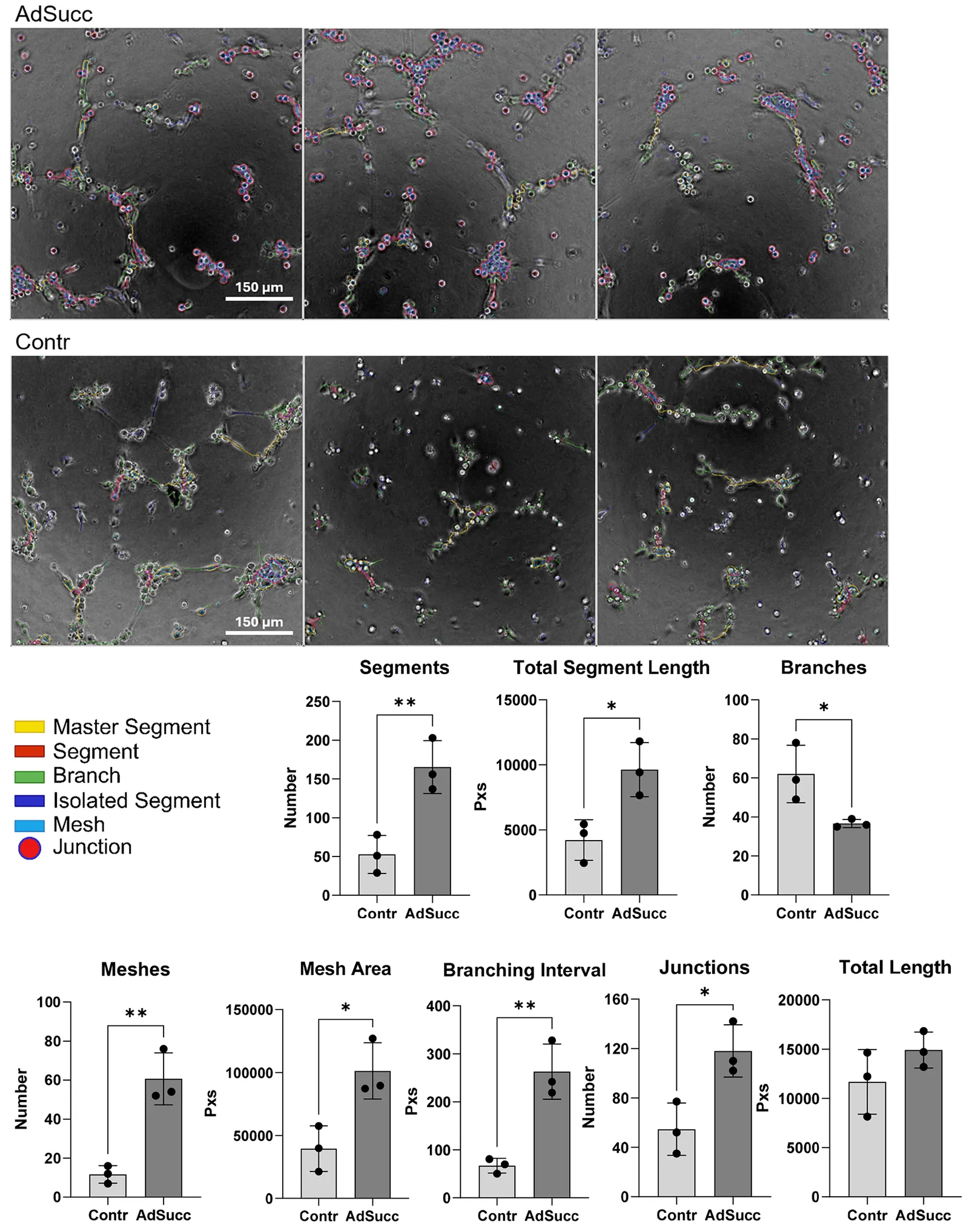
Adenylosuccinate (AdSucc) facilitates the formation of a tube-like network by human umbilical vein endothelial cells (HUVEC). HUVEC suspended in basal medium with 2% serum containing either 500 μM AdSucc or PBS (control, Contr) were plated at passage 3 (passage 1 after cryorecovery) onto basement membrane extract, and the tubular network was imaged using a phase-contrast microscope at 20x magnification. Analysis was performed using FIJI with the angiogenesis analyzer plugin. Nodes are pixels at branch points. Junctions are single or groups of touching nodes. Segments are portions between two junctions. Branches are connected to one junction but no other structures. Master segments lie between two junctions connected to more than one segment. Meshes are enclosed areas formed by segments or master segments. Data are presented as individual values and mean ± standard deviation (n = 3). *p*-values were determined by two-tailed, unpaired Student’s *t* test and are shown for all measurements, while *p*≤0.05 were considered statistically different.

To further validate AdSucc angiogenic properties, we next used *in vitro* tube formation model with cells of human origin. In this assay, HUVECs form tube-like structures when cultured on a matrix of BME ^30,31^. HUVECs exposed to 500 μM AdSucc for 8 h exhibited a robust pro-angiogenic response compared with vehicle-treated controls (Fig. 3). Quantitative image analysis revealed increases in network maturity and complexity. AdSucc facilitated tube formation through HUVEC adhesion to and modification of BME, evident from a 3.1-fold increase in the number of segments and total segment length and 41% decrease in the number of branches (segments not connected to another structure) ^32^. It also facilitated migration and cell-to-cell interaction demonstrated by a 3.9-fold increase in branching interval, 5.2-fold increase in number of meshes (area enclosed by structures), and 2.6-fold increase in total mesh area. The number of junctions (places where multiple tubes connect) was also 2.2-fold increased, indicating complex network formation in the presence of AdSucc. The total tube length increase was not statistically significant (*p*=0.2), likely due to high variability in the control set. Notably, AdSucc effect on tube formation was most pronounced in HUVECs used shortly after thawing from cryopreservation (passage 1) compared to well-recovered cells (data not shown). Because HUVECs are stressed shortly after removal from cryopreservation ^33^, this observation suggests that AdSucc preferentially enhances angiogenic capacity in stressed or compromised endothelial cells. This is important because AdSucc is increased under ischemia ^12^ when endothelial cells are under stressful/compromising conditions ^34,35^, indicating AdSucc potential role in the recovery from ischemic stroke or other conditions in which the endothelium is compromised.

**Figure 4.**
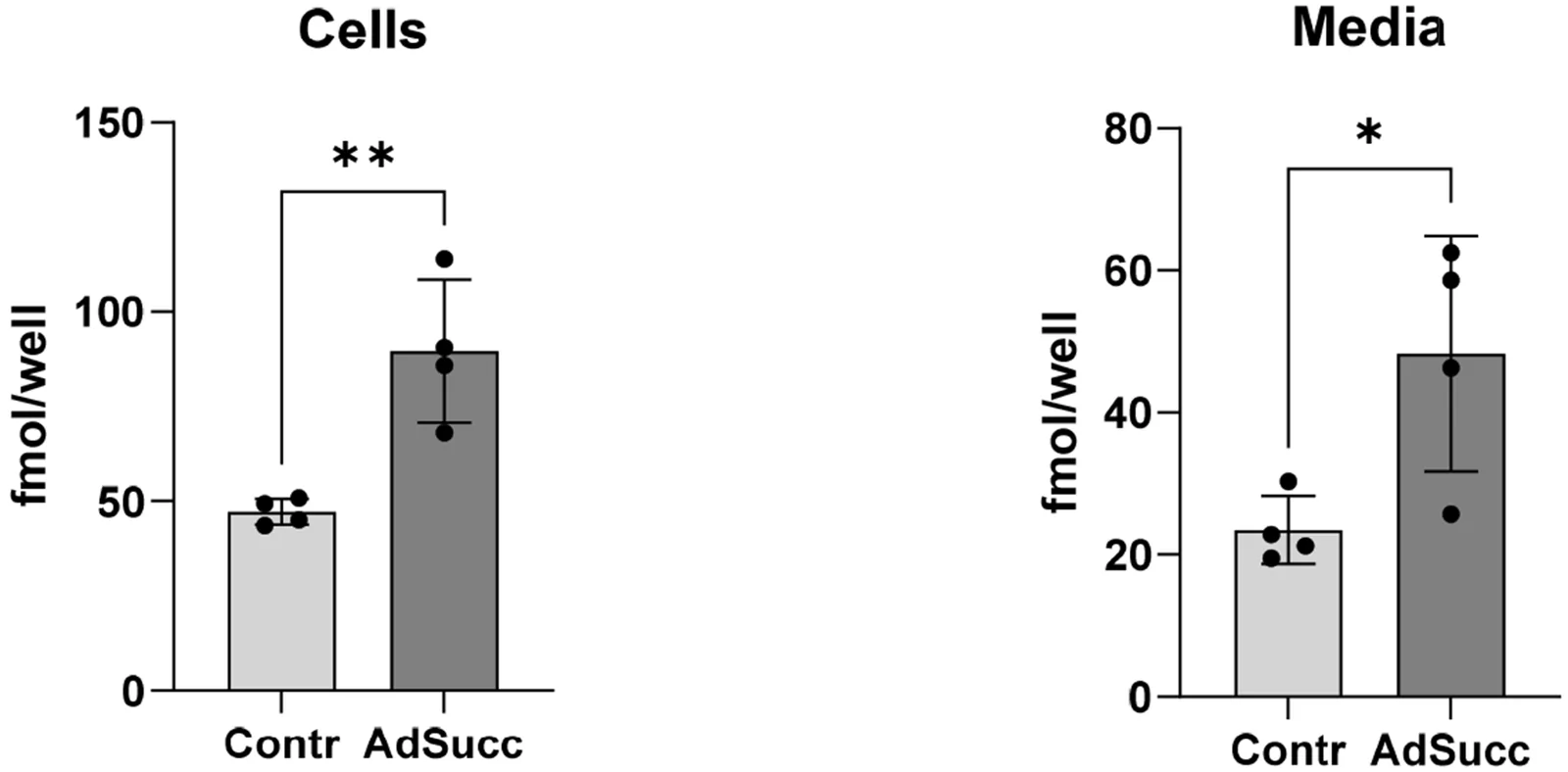
AdSucc stimulates cAMP production in primary human umbilical vein endothelial cells (HUVEC). cAMP levels were measured by ELISA in cell lysate (**A**) and media (**B**) collected from HUVECs treated with 500 μM AdSucc or PBS (control) for 8 h in serum-free media. (**C**): cAMP was measured in the serum free media with or without added AdSucc (N/D: not detected; * - *p* < 0.05; ** - *p* < 0.01 using two-tailed, unpaired Student’s *t* test).

To begin addressing the mechanism of AdSucc pro-angiogenic signaling, we measured its effect on cAMP, an important secondary messenger involved in angiogenesis ^36–39^. Treatment with 500 μM AdSucc for 8 h, the same conditions used in the tube formation assay, significantly increased cAMP levels in HUVECs, with 1.9- and 2.1-fold increases in cell lysates and conditioned media, respectively, relative to vehicle controls (Fig. 4). These results were not attributable to assay interference from AdSucc because cAMP signal was below detection limits in the presence of 500 μM AdSucc in the blank samples without cells. These data indicate, for the first time, that AdSucc stimulates both intracellular accumulation and extracellular release of cAMP in endothelial cells, providing additional support for AdSucc signaling properties. The ability of AdSucc to stimulate the production of this secondary messenger might outline one of the mechanisms underlying its pro-angiogenic properties, acting through established or novel pro-angiogenic receptors.

In summary, our data indicate a novel role for AdSucc as a pro-angiogenic signaling molecule. Further studies are required to validate the biological significance of AdSucc in promoting angiogenesis as an adaptation mechanism to low-energy conditions, and during recovery from ischemia-induced injury.

## Materials and Methods

### Animals

The handling and treatment of mice in this study were conducted in accordance with the National Institutes of Health Guidelines for the Care and Use of Laboratory Animals, a protocol approved by the University of North Dakota IACUC (protocols 2039-0056 and 2403-008), and in compliance with Animal Research: Reporting in In Vivo Experiments (ARRIVE) guidelines ^40^. Twenty-eight male and female (4-6 month old) Rag1^−^ mice (RRID:IMSR_JAX:002096, Jackson Laboratory) were randomly assigned to experimental groups. Rag1^−^ mice, which lack functional B and T lymphocytes were used in these experiments to minimize the influence of a strong, adaptive immune response on angiogenesis upon surgical angioreactor implantation ^41^. Mice were provided with a standard laboratory chow diet and water *ad libitum*. All experiments were conducted during the light cycle.

### Directed In Vivo Angiogenesis Assay (DIVAA)

For the *in vivo* angiogenesis assay, we used a reproducible quantitative mouse Directed *In Vivo* Angiogenesis Assay (DIVAA) approach as previously described ^42^. DIVAA utilizes subcutaneously implanted small (20 μL internal volume), semi-closed silicone cylinders (angioreactors) filled with an extracellular matrix extract containing the angiogenic compound – in our case, AdSucc and its metabolites. Sterilized silicone cylinders were filled with VitroGel hydrogel matrix without growth factors (VHM01, TheWell Bioscience, Monmouth Junction, NJ) containing either AdSucc (1 mM), AMP (20 μM), adenosine (Ade, 0.6 μM), or PBS and allowed to solidify for at least 1 h prior to implantation. In our pilot study, AdSucc was mixed with hydrogel and incubated for 7 days at 37 °C, producing no significant change in AdSucc concentration (data not shown), indicating AdSucc chemical stability under DIVAA conditions. Before implantation, mice were treated with sulfamethoxazole/trimethoprim (10/2 mg, per 100 ml drinking water) (Novitium Pharma LLC, East Windsor, NJ) one day prior to and continued for the duration of the assay. Angioreactors were implanted subcutaneously according to the manufacturer’s instructions under anesthesia with ketamine/xylazine (100/10 mg/kg, i.p.). Angioreactors containing AdSucc, AMP, or Ade were implanted in the contralateral (vs. PBS containing angioreactors) flanks of the mice. Ten days post implantation, mice were anesthetized with Isoflurane (5%) and euthanized *via* cervical dislocation. Angioreactors were surgically excised and tissue extending from the open end was removed. The closed end of the angioreactor was clipped and the hydrogel was removed by rinsing with 1ml VitroGel cell recovery solution (TheWell Biosciences, Monmouth Junction, NJ). Cell recovery was performed according to the manufacturer’s instructions and quantified using fluorescein-labeled *Griffonia simplicifolia* lectin I isolectin B4 (Vector Laboratories, Newark, CA) ^42^. Fluorescence was determined at 495/515 nm using a FlexStation 3 microplate reader (Molecular Devices, San Jose, CA). To characterize the metabolism of AdSucc in the angioreactors, we analyzed metabolites contained in the angioreactors by UPLC-MS at 0, 1, 3, 5, 10-days post-implantation.

To determine the role of AdSucc in the formation of functional perfused vessels, mice were implanted with angioreactors as described above. Ten days post-implantation, mice were anesthetized with 5% isoflurane and maintained at 2% isoflurane balanced with oxygen. Anesthetized mice were injected with Evan’s blue dye (4% in saline, injected 200 ul, retro-orbitally) and euthanized by cervical dislocation 15 min post-injection. Angioreactors were removed as described above. Contents of angioreactors were mixed with 30 μl of 50% trichloroacetic acid (w/v) in saline and subjected to sonication, followed by centrifugation at 10,000xg for 15 min at room temperature. Twenty μl of extract was transferred to a black-walled, clear-bottom 96-well microplate, and each well was supplemented with 30 μl of 100% ethanol to increase optic pathlength ^43^. Fluorescence was determined at 620/680nm against a standard curve using a FlexStation 3 microplate reader (Molecular Devices, San Jose, CA).

### LC-MS Analysis

DIVAA matrix were extracted and analyzed as previously described ^12^. Briefly, 20 μL of matrix was mixed with 200 μL of MeOH:water (3:1) with 4 μg ATP-^13^C_10_ and 4 μg glutamate (Glu)-^13^C_5_^15^N (Cambridge Medical Isotopes, Inc., Tewksbury, MA) as internal standards and subjected to sonication (3x 7sec at room temperature, sonic dismembrator with 4C15 probe, Fisher Scientific, Waltham, MA) followed by centrifugation at 10,000 x g for 15 min at room temperature. The supernatant was transferred to microinserts (MicroSolv, Wilmington, NC), and 10 μL extract was analyzed by ultra-high performance liquid chromatography- high resolution mass spectrometry (UPLC-MS).

Nucleotides and amino acids were resolved on a ZIC-pHilic column (5 μm, 150 × 2.1 mm; Millipore Corporation, Burlington, MA) with a ZIC-pHilic Guard cartridge (20 × 2.1 mm; Millipore Corporation, Burlington, MA) at room temperature. The UPLC system consisted of a Waters ACQUITY Premier UPLC pump and well-plate autosampler (Waters, Milford, MA) maintained at 30°C. The binary solvent system consisted of solvent A: water with 10mM ammonium bicarbonate and 0.2% ammonium hydroxide and solvent B: 100% acetonitrile. An LC gradient was as previously described ^12^.

The UPLC eluent was analyzed with a Waters SYNAPT XS quadrupole time-of-flight mass spectrometer (Waters, Milford, MA) operating in negative electrospray ionization mode. The mass analyzer was operated in MS^E^ and mass correction was applied using leucine enkephalin (Waters, Milford, MA) (100 pg/μL in ACN:water (1:1) containing 0.1% formic acid), as previously described ^12,44,45^. Instrument control and acquisition were performed using MassLynx v4.2 (Waters, Milford, MA) and peak integration was performed using QuanLynx software utilizing a 0.02 ppm mass window. AdSucc, AMP, IMP, and urate were quantified against ATP-^13^C_10_ internal standard (2 μg/sample), Ade was quantified against Ade-^13^C_5_ (20 ng/sample), and Fum was quantified against Glu-^13^C_5_^15^N internal standard (2 μg/sample) using corresponding calibration curves.

### Cell Cultures

Human umbilical vein endothelial cells (HUVECs) were obtained from the American Type Culture Collection (Item No. PCS-100-013, ATCC, Manassas, VA, USA) at passage 2 and cultured in VascuLife^®^ VEGF endothelial medium complete kit (Item No. LL-0003, Lifeline Cell Technology, Frederick, MD, USA) without the addition of antibiotics at 37 °C in a humidified incubator with 5% CO_2_. For cAMP measurement, cells were used at passage 4 (passage 2 after cryorecovery) and grown to ~80% confluence in a 96-well plate prior to treatment. HUVECs were treated with AdSucc at a final concentration of 500 μM for 8 hours in media without serum or growth factors. Vehicle-treated cells served as controls. For tube formation, HUVECs at passage 3 (passage 1 after cryorecovery) were used.

### Tube Formation Assay

HUVECs were seeded at a density of 10,000 cells per well into an ibidi μ-Plate 96 well 3D (Item No. 89646, ibidi USA Inc, Fitchburg, WI, USA) after the inner well was filled with 10 μL of Cultrex Reduced Growth Factor Basement Membrane Extract (Item No. 3433-010-01, R&D Systems, Minneapolis, MN, USA). Cells were plated in 50 μL basal medium supplemented with 2% fetal bovine serum with either AdSucc with pH adjusted to 7.5 in PBS (Item No. 29696, Cayman Chemical, Ann Arbor, MI, USA) or phosphate-buffered saline (pH=7.5) as a control. The plate was placed in a stage-top incubator maintained at 5% CO_2_ and 100% humidity at 37 °C. Cells were imaged using phase-contrast microscopy with a Leica DMi8 Thunder imaging system (Leica Microsystems Inc, Deerfield, IL, USA) at 20X magnification at 8 h.

Analysis was performed using ImageJ software with the Angiogenesis Analyzer plugin ^46^. Prior to analysis, contrast was enhanced (Enhance Contrast, saturated=0.35), images were converted from 16 bit to RGB color, and a Gaussian blur (1 pixel radius) was applied. Angiogenesis Analyzer creates and measures a map of the structures of tube formation. Nodes are pixels at branch points. Junctions are single or grouped touching nodes. Segments are portions between two junctions. Branches are connected to one node but no other structures. Master segments lie between two junctions connected to more than one segment. Meshes are enclosed areas formed by segments or master segments ^47^. Angiogenesis Analyzer plugin settings were as follows: Minimum object size: 30 pixels; Minimum branch size: 40 pixels; Artifactual loop size: 850 pixels; Isolated element size threshold: 40 pixels; Master segment size threshold: 150 pixels; Iteration number: 3.

### cAMP Analysis

Intracellular and extracellular cAMP levels were quantified using the Cyclic AMP Select ELISA kit (Item No. 501040; Cayman Chemical, Ann Arbor, MI, USA) according to the manufacturer’s instructions. Briefly, after treatment, cells were placed on ice. The media was removed, centrifuged to remove debris, and used for cAMP extracellular analysis. For intracellular cAMP, cells were collected in 100 μL of the supplied ELISA buffer, probe sonicated, and centrifuged to remove debris. cAMP concentrations were calculated from a standard curve. Importantly, cAMP signal was below detection limits in the presence of 500 μM AdSucc in the blank samples without cells, indicating no cross-reactivity between cAMP and AdSucc under the assay conditions.

### Statistical Analysis

For pairwise comparison, we used a two-tailed, unpaired Student’s *t*-test without correction for multiple comparisons. Statistical analysis comparing multiple groups was performed using a one-way ANOVA with Tukey’s *post hoc* test. The differences in the DIVAA assay were determined using a two-tailed, paired Student’s *t*-test. For presentation of DIVAA assay data, relative fluorescent units were normalized against the mean of the control. This normalization method did not alter *p*-value. Values were considered significant with *p*≤0.05 and are expressed as mean±SD. Statistical analyses were performed using GraphPad Prism 10 (GraphPad, San Diego, CA).

## Data Availability

All data are contained within the manuscript

## Abbreviations

Ade: adenosine
AMP: adenosine monophosphate
AdSucc: adenylosuccinate
DIVAA: directed *in vivo* angiogenesis assay
Fum: fumarate
Glu: glutamate
HUVEC: human umbilical vein endothelial cells
IMP: inosine monophosphate
PBS: phosphate-buffered saline
PNC: purine nucleotide
TCA: tricarboxylic acid cycle
